# The intracranial compartmental syndrome: a proposed model for acute brain injury monitoring and management

**DOI:** 10.1186/s13054-023-04427-4

**Published:** 2023-04-10

**Authors:** Daniel Agustín Godoy, Sérgio Brasil, Corrado Iaccarino, Wellingson Paiva, Andres M. Rubiano

**Affiliations:** 1 Sanatório Pasteur, Catamarca, Argentina; 2 Experimental Surgery Laboratory and Division of Neurological Surgery, University of São Paulo Medical School, Av. Eneas de Carvalho Aguiar 255, Sao Paulo, Brazil; 3 Department of Biomedical, Metabolic and Neural Sciences, University Modena and Reggio Emilia, Modena, Italy; 4 Department of Neurosurgery, University Hospital of Modena, Modena, Italy; 5 Emergency Neurosurgery, AUSLRE IRCCS, Reggio Emilia, Italy; 6 Universidad El Bosque. Bogotá, Bogotá, Colombia; 7 MEDITECH Foundation, Cali, Colombia

**Keywords:** Intracranial compartmental syndrome, Intracranial hypertension, Cerebral compliance, Intracranial pressure waveform, Acute brain injury, traumatic brain injury

## Abstract

For decades, one of the main targets in the management of severe acute brain injury (ABI) has been intracranial hypertension (IH) control. However, the determination of IH has suffered variations in its thresholds over time without clear evidence for it. Meanwhile, progress in the understanding of intracranial content (brain, blood and cerebrospinal fluid) dynamics and recent development in monitoring techniques suggest that targeting intracranial compliance (ICC) could be a more reliable approach rather than guiding actions by predetermined intracranial pressure values. It is known that ICC impairment forecasts IH, as intracranial volume may rapidly increase inside the skull, a closed bony box with derisory expansibility. Therefore, an intracranial compartmental syndrome (ICCS) can occur with deleterious brain effects, precipitating a reduction in brain perfusion, thereby inducing brain ischemia. The present perspective review aims to discuss the ICCS concept and suggest an integrative model for the combination of modern invasive and noninvasive techniques for IH and ICC assessment. The theory and logic suggest that the combination of multiple ancillary methods may enhance ICC impairment prediction, pointing proactive actions and improving patient outcomes.

## Introduction

For years, the evaluation and management of intracranial hypertension (IH), based on specific thresholds, have been the main target ("tip of the iceberg") for the treatment of acute brain injury (ABI), especially for traumatic brain injury (TBI) [1]. The increase in intracranial pressure (ICP) generates deleterious effects because of the displacement of anatomical structures, leading to a cascade of brain swelling, ischemia and generating different degrees and types of brain tissue herniation [2, 3].

Recently, an expert panel developed management algorithms for TBI care based on 22 mmHg for ICP threshold [4, 5]. Notwithstanding, such recommendations are supported only by lower evidence levels [6, 7]. In fact, the only randomized controlled trial for the management of TBI comparing ICP monitoring vs a clinical protocol guided by examination and neuroimaging (Best-Trip trial) demonstrated that ICP monitoring was not a necessary intervention when serial bedside neurological examination and brain imaging were taken [8]. This study changed the paradigm regarding the consideration of ICP as an isolated central intervention in TBI [9]. Moreover, it led to the emergence of arguments advocating against maintaining an empirical, fixed and rigid ICP cutoff as a pillar for starting different medical and/or surgical interventions [7, 10].

The secondary events after ABI are heterogeneous between subjects [11]. Moreover, even for the same patient, adjusting ideal cerebral perfusion may require arterial blood pressure (ABP) changes during periods of physiological instability following injury [11, 12]. Hence, determining the most vulnerable periods for tissue hypoxia and cellular dysfunction [13, 14] can be challenging [15–18]. In this context, intracranial compliance (ICC) impairment, the threshold with which intracranial volume has overpassed the inner compensatory reserve [19, 20], can be a more reliable target than ICP alone [21].

The recent advances in technology have brought intensive care units the opportunity to monitor closely and predict the undesired consequences of ICC impairment [22, 23], with a synergist diagnostic potential when these techniques are combined [24, 25]. Therefore, the modern management of IH should focus on a different and more integrated perspective, considering the instruments available to monitor these phenomena [26–28].

The present perspective review aims to propose the integration of monitoring techniques currently available to assess ICC impairment, such as ICP monitoring, transcranial Doppler (TCD), pupillometry, brain oximetry, near-infrared spectroscopy (NIRS), optic nerve sheath diameter ultrasound (ONSD) and automated ICP waveform analysis (ICPW). With these tools at hand, we propose a model and treatment algorithm utilizing the intracranial compartmental syndrome (ICCS) that may serve as an improvement in IH management. Mapping how different techniques can be associated is particularly important among locations where resources are scarce, such as low-income countries.

## The intracranial compartmental syndrome

### Definition

**Fig. 1:**
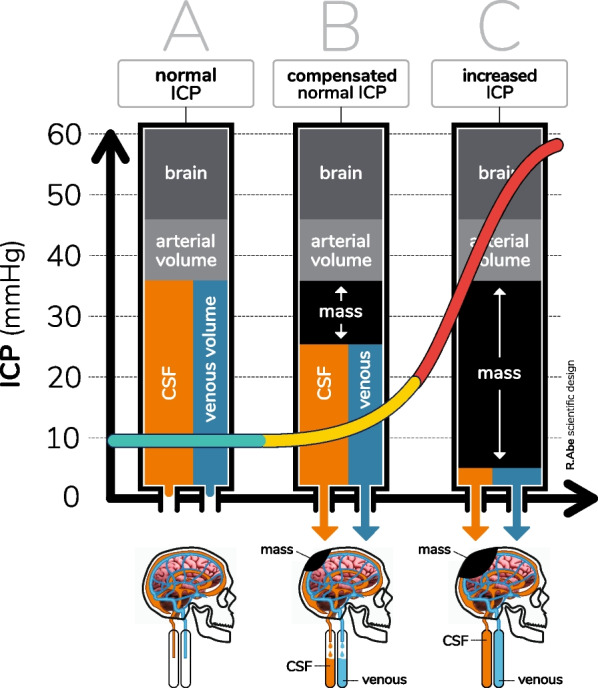
Different phases of the compensatory system. In the first phase (**a**), compensatory system is effective during a mass expansion. ICP does not change in this early phase, being ICC and the compensatory system adjusted. In a second phase (**b**), the compensatory system starts to fail following more increase in the mass effect. CSF and veins outflow are starting to be overloaded, beginning brain deformation and ICC impairment. In a third phase (**c**), the compensatory system is completely exhausted, and brain deformation and loss of ICC are evident. ICP: intracranial pressure, ICC: intracranial compliance, CSF: cerebrospinal fluid. Adapted from Wykes et al. [31]

The ICCS is an ICC impairment diagnostic model applying different monitoring techniques with educational purpose as a standard of care. As for IH, ICCS occurs because the skull is a non-extensible compartment with limited adaptation to changes in pressure, and when the inner volume reaches a critical level, ICC is exhausted (Fig. 1) [20, 29–31]. The rigid cranial cavity is interconnected with other cavities as thorax and neck by venous and cerebrospinal fluid (CSF) systems, so IH may develop because of cranial and extracranial conditions (Table 1). As for any body cavity, when the inner pressure increases severely, it causes hypoperfusion, ischemia and tissue damage as a consequence of sensitive structures compression, such as nerves and blood vessels [32–34].

**Table 1:** Causes of intracranial hypertension

**1. Intracranial**
Extrinsic compression
Depressed skull fractures
Subdural, extradural hematomas
Cerebral contusions
Cerebral edema
Thrombosis
**2. Extracranial**
• Cervical collars
Neck lateralization
Jugular thrombosis (central line, SVjO_2_ monitoring devices)
Orotracheal tube tethering
• Thorax (intrathoracic pressure increase)
Pneumothorax
Hemothorax
Mechanical ventilation
Inadequate PEEP levels
Airway obstruction
Thrombosis (central line)
Severe pulmonary embolism
Asynchrony with mechanical ventilator
• Abdomen (intrabdominal pressure increase)
Fluid Resuscitation
Ileus
Gastroparesis
Pneumoperitoneum
Hemoperitoneum

*PEEP* positive expiratory end pressure, *SVjO*_*2*_ jugular bulb oxygen saturation

**Fig. 2:**
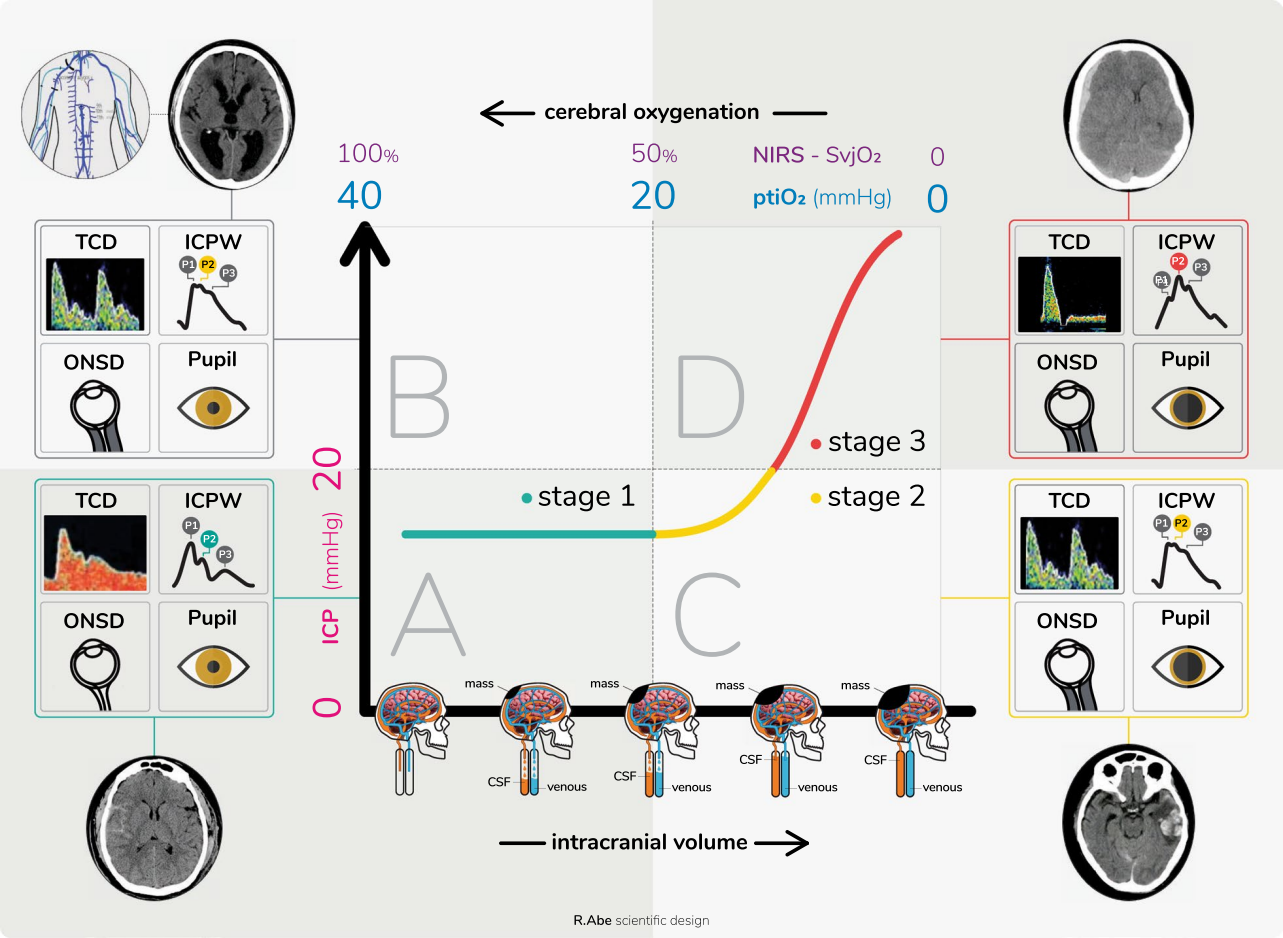
Proposed integrative model. Stage 1: normal ICC, stage 2: ICC impairment and stage 3: severe ICC impairment. Types A-D explained in detail in the text. ICP: intracranial pressure. ICC: intracranial compliance, ICPW: ICP waveform, NIRS: near-infrared spectroscopy, PtiO_2_: cerebral oximetry

In early stages of ICCS development, a space-occupying lesion (contusion) or an increase in brain parenchyma volume (edema) does not cause an increase in ICP, so long as the compensation systems and cerebral autoregulation work [3]. If the process is not aborted at this time, ICP will increase exponentially, compromising perfusion, oxygenation, energy usage and creating compartmental gradients that will anatomically distorting brain tissue. It is important to remark that these changes are not necessarily associated with specific ICP number thresholds, as we can find patients with loss of ICC within a predetermined “normal range” of ICP or in patients with preserved ICC that demonstrate ICP above these thresholds (Fig. 2).

**Fig. 3:**
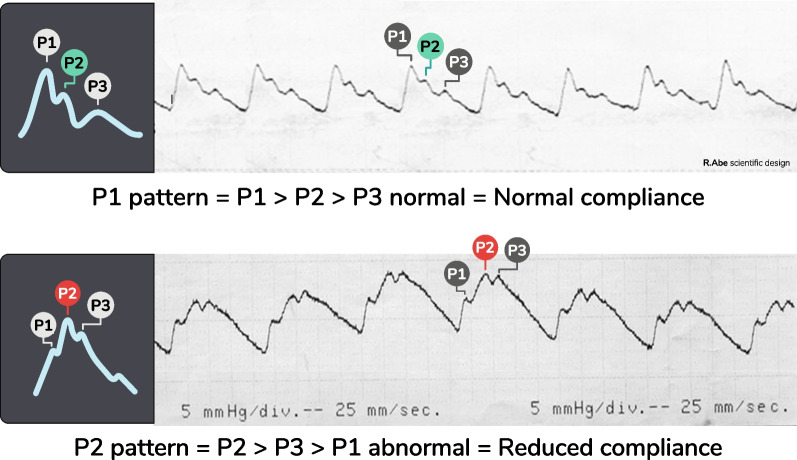
ICP waves registered at 25 mm per second showing the three components (P1, P2 and P3). **a** Normal pattern; **b** pattern of impaired compliance. ICP: intracranial pressure (Source: authors)

Therefore, the hallmark of reduction in ICC must rely on the ICP pulse morphology or waveform (ICPW) [35]. ICPW has been extensively studied and constitutes the leading monitor in the ICCS diagnostic toolbox. The changes in ICP pulse morphology have been directly linked to ICC impairment, especially when the second peak (P2) assumes a higher amplitude than the first peak (P1, Fig. 3), forecasting IH [36, 37]. In combination with changes in ICPW, other invasive and noninvasive methods can be added as synergistic adjuncts to monitor brain oxygenation, compliance and blood dynamics.

### Thresholds

ICP probes may display a spectrum of measured values, from exclusively ICP mean values up to ICP trends, systolic and diastolic values, brain temperature and waveforms [38]. Without an automated analysis of ICPW from invasive methods, the contour analysis relies on subjectivity and expertise to conclude when P2 surpasses P1 in amplitude [39]. Oximetry probes may also provide brain temperature and a local (around 2.5 cc) brain tissue oxygenation (PbtO_2_), with ideal around 20–35 mmHg [40], whereas NIRS and jugular venous oxygen saturation (SvjO_2_) provide percentages of hemoglobin oxygen saturation. For ICCS, we used ICP > 20 mmHg and PbtO_2_ < 20 mmHg [41] or SO_2_ < 50% if NIRS or SvjO_2_ are used [42].

An ICU validated, noninvasive and mobile technique to monitor ICP variations based on ICPW has been recently entered the market (Braincare Corp, São Carlos, Brazil) [38, 43–46]. The system is based on cranial micrometric deformation; it currently does not provide ICP values, but extracts parametric values from the pulse slopes that are correlated with IH [38]. For ICCS, we applied the P2/P1 ratio > 1.2 provided by this technique as indicator of IH [44].

Ultrasound techniques, such as duplex and TCD, can be useful in several neurovascular diagnostic areas: cerebral autoregulation assessment [3], embolic activity [47], arterial [48] and venous stenosis, as well as supportive evidence in brain death [49]. TCD acquires waveforms derived from blood velocities and may indicate reduction in cerebral perfusion pressure through dedicated software [22], the pulsatility index (PI) and/or reduction in mean velocities [25]. Duplex also can observe intracranial hematomas and middle line shift [50], and evaluate the ONSD. It has also demonstrated excellent negative predictive value for the estimation of ICP [51]. For ICCS, we used the PI > 1.2 and ONSD > 5 mm as indicators of IH [52].

Pupillometry can provide sedation status, assessment of pain, prediction of clinical deterioration and outcome [53]. Although it has reduced capacity to detect IH by the pupillary reflex alone, the automated neurological pupil index (NPi) present in dedicated manufacturer (NeurOptics, Irvine, USA) is reliable to observe worsening in neurological condition as consequence of IH, when serial measures are performed [53]. For ICCS, we used the NPi < 3 as an indicator of neurological deterioration. Main techniques advantages and limitations included in the model are summarized in Table 2.

**Table 2:** Characteristics of most relevant noninvasive surrogate techniques for ICP monitoring

	TCD	ONSD	Pupillometry	Brain4care
Use Mode	Serial/continuous	Serial	Serial	Serial/continuous
IH estimation	Numeric	Y/N	Y/N	Y/N
Operator training	High	Low	Low	Low
Operator dependence	High	High	Low	Low
Strengths	Multiple different vascular diagnostics	Readiness to obtain results, easily repeatable	Low operator dependence, assessment of pain in sedated patients	High negative predictive value, monitoring during interventions
Weaknesses	Depends on acoustic windows and operator availability	High interobserver variation	Low accuracy for ICP estimation	Not suitable for highly agitated patients, neurosurgery leads to thresholds shift

*ICP* intracranial pressure, *IH* intracranial hypertension, *ONSD* optic nerve sheath diameter ultrasound, *TCD* transcranial Doppler, *Y/N* yes or not

EEG has not been included, but should be considered as an additional information to this ICCS algorithm. Furthermore, metabolic crisis [54] and spreading depolarizations [55] are examples of real menaces for ABI patients that can occur unnoticed in this model [56], being a limitation of ICCS.

## Proposed diagnostic model

**Fig. 4:**
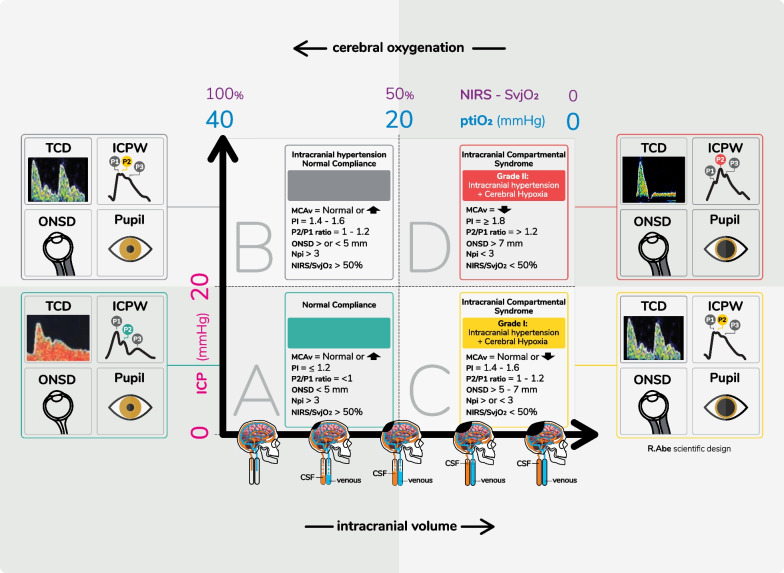
Integrative model of multimodal monitoring with the thresholds for different invasive and noninvasive techniques. Types A-D explained in detail in the text. ICP: intracranial pressure. ICPW: ICP waveform, NIRS: near-infrared spectroscopy, PtiO_2_: cerebral oximetry

The proposed model integrates the monitoring of ICC through the analysis of ICPW to the traditional invasive and/or noninvasive monitoring methods of ICP and cerebral tissue oxygenation (Fig. 4). The techniques included have a solid and compelling rationale for their use, despite the fact that large trials remain lacking [57, 58]. As many of these techniques are referred to physiological phenomena, it is not expected that all techniques show their results exactly as presented below, but inconclusive results may guide continuous/serial monitoring and revisiting the patient records. Considering the variety of resources from one location to another, as well as the advantages and limitations of each technique, this is not a condition to be assessed exclusively if all methods are available. Rather, it is a recommendation for practitioners to take hand of all resources present; the more the information available, the more likely an assertive decision will result. Of course, the comprehensive management of ICCS depends on a thorough review of the medical record and available brain imaging.

Four evolutionary patterns are proposed, utilizing the ICPW characterization:

**Type A:** normal pattern. Absence of intracranial hypertension and tissue hypoxia on invasive and noninvasive monitoring, i.e., preserved ICPW.

**Type B:** IH without impairment of cerebral oxygenation while maintaining ICC (borderline ICPW). Such a pattern may be seen in insidious chronic conditions such as obesity [59], in the early stages of hydrocephalus development [60] or during the early stages of space-occupying lesions or cerebral edema [61]. Furthermore, extracranial causes of increased ICP (pneumothorax, mechanical ventilator asynchrony [62, 63], airway obstruction, intrabdominal hypertension [64]).

**Type C:** Grade I ICCS. Alteration of ICC evidenced through the change in the ICP pulse morphology (P2 > or = P1), in the *“absence of an increase in the numerical value of the ICP*.” Causes of this pattern are temporal or frontal contusions < 25 cc (diffuse injury type II of the Marshall’s tomographic classification) or laminar extra-axial lesions that do not cause midline deviation. Depending on the evolutionary stage, it may or may not be accompanied (advanced) by brain tissue hypoxia (early).

**Type D:** Grade II ICCS. The syndrome in its fullness, as a life-threatening condition. It is characterized by total loss of compliance with the presence of marked morphological changes in the ICPW with effacement of the P1 component and adoption of a pyramidal shape, accompanied by IH and cerebral tissue hypoxia. Pattern that can be observed in non-evacuated space-occupying lesions > 25 cc or diffuse type III or IV injury of the Marshall’s classification.

## Therapeutical approach

Following the pathophysiological reasoning of the proposed model, the therapeutic approach can be based on the following premises:

**Type A:** Treatment will be based on clinical, imaging, hemodynamic, metabolic and intracranial oxygenation monitoring. From their analysis, the intensivist will proceed to the implementation of general care measures, which may include physiological neuroprotection, mechanical ventilation and sedation/analgesia, avoiding secondary insults, and preventing deep vein thrombosis, gastrointestinal bleeding and infectious complication [65]. Anticonvulsants when indicated, early nutrition and rehabilitation are important additional measures. This global approach should be continuous even for the other subtypes [4, 6, 65, 66].

**Type B:** IH without ICC impairment. Before starting therapy, it is important to carry out an exhaustive analysis of the cause (whether intra- or extracranial, for example), since the therapy will depend on etiology [66]. In case of hydrocephalus, external ventricular drainage will be the choice; while if the origin is increased intrathoracic pressure, due to asynchrony with mechanical ventilation, deepening sedation/analgesia after analysis of the ventilatory mode will be priorities.

**Type C:** ICCS grade I. IH may or may not be present, but ICC impairment leads to brain tissue oxygenation alteration. This situation is probably the most difficult to defining therapeutics. Although initial medical management of IH and cerebral tissue hypoxia is based on individual institutional guidelines or international consensus [4, 66, 67], it is essential to keep in mind the following premises: (a) close, continued follow-up monitoring and further therapeutic response based on wave morphology [68, 69]; (b) refrain from escalation of any medical treatment beyond recommended levels, if the response to that intervention is not satisfactory; and (c) early consideration of CSF drainage and/or surgical evacuation of space-occupying injuries or decompression of the cranial cavity.

**Type D:** ICCS grade II. The syndrome is fully developed. Combined medical and surgical therapy is mandatory, but prompt consideration of the latter is considered essential, since decompression of the cranial cavity is urgently necessary independent of the nature (whether focal or diffuse) of the lesions [70, 71].

## Conclusions and future perspectives

Modern management of ABI has broken the simplistic intracranial hypertension-based model of care. Other phenomena such as brain tissue hypoxia and energy dysfunction are important to recognize, prevent and treat to optimize results. Severe TBI is dynamic and heterogeneous. The advancement and analysis of multimodal monitoring brought with it the concept of “personalized therapy.” The proposed model integrates the monitoring of intracranial compliance with the traditional monitored variables (invasive or noninvasive) during severe TBI. The ICCS is defined not by a specific numeric threshold of ICP monitoring, but based on its subtype and multimodal monitoring, suggesting therapeutic approaches for emergency conditions. Large-scale studies are necessary to evaluate this proposed model in addition to validate the use of new noninvasive monitoring techniques for understanding these new concepts. We advocate for moving toward new concepts and paradigm shifts in the management of ABI in order to decrease mortality and disability associated with a delayed decision-making process.

## Data Availability

Not applicable.

## Abbreviations

TBI: Traumatic brain injury
ICP: Intracranial pressure
ICPW: Intracranial pressure waveform
CSF: Cerebrospinal fluid
MLS: Midline shift
CT: Computed tomography
IH: Intracranial hypertension
ICCS: Intracranial compartmental syndrome
CBV: Cerebral blood volume
ICC: Intracranial compliance
CPP: Cerebral perfusion pressure
MAP: Mean arterial pressure
TCD: Transcranial Doppler
NPV: Negative predictive value
B4C: Brain4care
TTP: Time to peak
ABI: Acute brain injury
